# Isolation and Characterization of Lytic *Proteus* Virus 309

**DOI:** 10.3390/v14061309

**Published:** 2022-06-15

**Authors:** Joshua Aaron, Leonardo J. van Zyl, Leon M. T. Dicks

**Affiliations:** 1 Department of Microbiology, Stellenbosch University, Stellenbosch 7600, South Africa; 19463146@sun.ac.za; 2 Institute for Microbial Biotechnology and Metagenomics, University of the Western Cape, Bellville 7535, South Africa; vanzyllj@gmail.com

**Keywords:** *Novosibovirus*, bacteriophage, *Proteus mirabilis*, genome annotation

## Abstract

*Proteus mirabilis* is frequently associated with complicated urinary tract infections (UTIs) and is the main cause of catheter-associated urinary tract infections (CAUTIs). Treatment of such infections is complicated and challenging due to the biofilm forming abilities of *P. mirabilis*. If neglected or mistreated, infections may lead to life-threating conditions such as cystitis, pyelonephritis, kidney failure, and bacteremia that may progress to urosepsis. Treatment with antibiotics, especially in cases of recurring and persistent infections, leads to the development of resistant strains. Recent insights into phage therapy and using phages to coat catheters have been evaluated with many studies showing promising results. Here, we describe a highly lytic bacteriophage, *Proteus*_virus_309 (41,740 bp), isolated from a wastewater treatment facility in Cape Town, South Africa. According to guidelines of the International Committee on Taxonomy of Viruses (ICTV), bacteriophage 309 is a species within the genus *Novosibovirus*. Similar to most members of the genus, bacteriophage 309 is strain-specific and lyse *P. mirabilis* in less than 20 min.

## 1. Introduction

*Proteus mirabilis* is a Gram-negative motile rod that belongs to the family Enterobacteriaceae and is widely distributed in soil and aquatic environments. The species is regarded as an opportunistic pathogen with inherent virulence properties such as swarming [1], production of hemagglutinins [2], fimbriae [3,4], urease [5], and capsular polysaccharides [6]. These are all characteristics enabling *P. mirabilis* to form biofilms on epithelial cells or artificial surfaces [7]. In addition, many strains of *P. mirabilis* have a range of antibiotic efflux pumps [8]. Cells in biofilm are also protected from the host’s immune system [9].

Most persistent and recurring urinary tract infections (UTIs) are caused by *P. mirabilis* [9,10] and urinary catheters have been identified as one of the major sources, referred to as catheter associated UTIs or CAUTIs. The latter may lead to severe bladder and kidney infections, the formation of kidney stones and cysts, pyelonephritis, permanent renal damage, bacteremia, and sepsis [9,10,11]. Treatment of infections is challenging as many strains are resistant to polymyxins, nitrofurans, tigecycline, and tetracycline [12,13]. Some *P. mirabilis* strains produce several β-lactamases (ESBLs) [14] carbapenemases [15] plasmid-mediated quinolone resistance genes (*qnr*D and *qnr*C) [16,17].

An alternative strategy of treatment is the use of bacteriophages, known as phage therapy [18,19,20]. The advent of next generation sequencing and virome studies have sparked renewed interest in phage research with frequent identification of new species being used as antimicrobial agents. Lytic phage cocktails have been developed for treatment of infections caused by ESCAPE pathogens (*Enterococcus faecium*, *Staphylococcus aureus*, *Clostridium difficile*, *Acinetobacter baumannii*, *Pseudomonas aeruginosa* and *Enterobacteriaceae* species) [21,22]. A recent study [23] has shown that a combination of bacteriophage KS12 and a low dose of meropenem inhibited the proliferation of *Burkholderia cenocepacia* in *Galleria mellonella* larvae. Treatment of a *Staphylococcus aureus* biofilm with bacteriophage PYO rendered the cells more sensitive to tetracycline and ciprofloxacin [24]. A combination of bacteriophage VB_KpnM_P-KP2 and gentamicin proofed effective in the elimination of *Klebsiella pneumonaie* serotype K47 [25]. Destruction of biofilms using bacteriophages with depolymerase activity showed promising results [20,26]. Examples are bacteriophages KTN6 and KTN28 that prevented *Pseudomonas aeruginosa* to form biofilms [27] and a combination of bacteriophages from the families *Podoviridae*, *Microviridae*, *Corticoviridae*, *Tectiviridae*, *Leviviridae,* and *Cystoviridae* that removed MDR biofilm-forming strains of *P. mirabilis* from glass beads [28]. *P. mirabilis* biofilm formation on catheters could be controlled for up to 168 h by pre-treating surfaces with a layer of podovirus vB_PmiP_5460 and myovirus vB_PmiM_5461 [29]. In another study [30], the blocking of urinary catheters by *P. mirabilis* could be controlled by releasing a combination of bacteriophages from a pH-responsive polymer [poly (methyl methacrylateco-methacrylic acid] (EUDRAGITsS 100). Here, we report on a bacteriophage, *Proteus*_virus_309, belonging to the genus *Novosibovirus*, with lytic activity towards a clinical isolate of *P. mirabilis* B16.

## 2. Materials and Methods

### 2.1. Bacterial Strains, Media, and Culturing Conditions

Clinical bacterial isolates (Table S1) were obtained from Tygerberg Hospital (Cape Town, South Africa) and their identity confirmed by partial 16S rDNA sequencing, using primers F8 (CACGGATCCAGACTTTGATYMTGGCTCAG) and R1512 (GTGAAGCTTACGGYTAGCTTGTTACGACTT). All chemicals, reagents, and growth media were obtained from Merck (Darmstadt, Germany) and ThermoFisher Scientific (Waltham, MA, USA). Bacteria were routinely cultured in Brain Heart Infusion (BHI) at 37 °C on an orbital shaker (100 rpm), or on BHI agar (1.2%, *w*/*v*, agar). Agar overlays and phage enrichment were performed using soft BHI agar (0.8%, *w*/*v*, agar).

### 2.2. Phage Isolation and Purification

Phages were isolated from the Athlone wastewater treatment plant (Cape Town, South Africa), using the enrichment protocol previously described [31]. Wastewater was centrifuged (8000× *g*, 15 min, 4 °C), the supernatant filtered through 0.2 µm GVS filters (Lasec, Cape Town, South Africa), and 1.0 mL inoculated into a 100 mL culture of *P. mirabilis* B16 in exponential phase, supplemented with 1 mM CaCl_2_. After 16 h of incubation at 37 °C, cells were harvested (10,000× *g*, 20 min, 4 °C) and the cell-free supernatant filtered through a sterile nitrocellulose filter (2.0 µm pore size). Phages were enumerated on soft BHI agar imbedded with *P. mirabilis* B16 as described by Anderson et al. [32]. The phage lysate was serially diluted (10^−1^ to 10^−10^) and 100 µL of each dilution inoculated into 5.0 mL BHI soft agar. The phage-host suspension was poured over BHI agar (1.2%, *w*/*v*, agar) and the plates incubated for 16 h at 37 °C. Plaques were selected, streaked onto BHI agar (1.2%, *w*/*v*) and 5 mL soft BHI agar, containing 100 µL *P. mirabilis* B16, poured over the streaks as per Cross et al. [33]. Plates were incubated for 16 h at 37 °C. This was repeated four times to obtain a pure phage population. Plaque forming units (PFU) was recorded and the lysate stored at 6 °C in phage buffer (10 mM Tris, pH 7.5, 10 mM MgSO_4_, 60 mM NaCl) or at −80 °C in 25.0% (*v*/*v*) glycerol.

### 2.3. Phage Concentration

Several phage lysate stocks were concentrated as described by Yamamoto et al. [34]. The efficiency of plating was determined allowing for confluent lysis on a standard BHI overlay plate. Twenty confluent lysis plates (90 mm) were incubated for 16 h at 37 °C, plates were flooded with 5.0 mL phage buffer and incubated for 2 h at 4 °C on an orbital shaker (50 rpm). Aspirated phages were collected from the plates, bacterial debris was centrifuged (10,000× *g*, 20 min, 4 °C), and the phage lysate filtered as described elsewhere. Phages were collected by adding polyethylene glycol (PEG8000, Sigma Aldrich, St. Louis, MO, USA) to a final concentration of 20% (*v*/*v*) and stored upright for 16 h at 4 °C. The following day, phages were harvested (20,000× *g*, 1 h, 4 °C) and the supernatant removed. The pellet was gently resuspended in 5.0 mL phage buffer and stored at 6 °C until used.

### 2.4. Transmission Electron Microscopy (TEM)

Ten microliters of the phage suspension (>1 × 10^10^ PFU/mL) was stained with 2.0% (*w*/*v*) uranyl acetate and pipetted onto carbon-coated copper grids (Agar Scientific, Stansted, UK) that were discharged using a EMS100 Glow Discharge Unit (Electron Microscopy Sciences, Hatfield, PA, USA). Stained samples were viewed using a FEI Tecnai F20 transmission electron microscope (ThermoFisher Scientific, Eindhoven, The Netherlands), fitted with a DE-16 camera (Direct Electron, San Diego, CA, USA). Images of the phages were taken at the University of Cape Town’s Electron Microscopy Unit (Cape Town, South Africa).

### 2.5. Extraction of Phage DNA

DNA was extracted from concentrated phage suspensions using the method of Göller et al. [35] but modified. Concentrated phage lysate (500 µL) was treated with 2.0 µL DNAse 1 (2000 U/mL) and 20.0 µL RNAse A (10.0 mg/mL), and incubated at 37 °C for 2 h. Fifty microliters of 0.5 M ethylenediaminetetraacetic acid (EDTA), 5.0 µL of proteinase K, and 50.0 µL of 10% (*w*/*v*) sodium dodecyl sulphate (SDS) were added, followed by 1 h incubation at 55 °C. The phage lysates were divided into two 2-mL microcentrifuge tubes to which 500 µL of phenol/chloroform/isoamyl alcohol (PCI; 25:24:1) was added and centrifuged (12,000× *g*, 5 min, 16 °C). The top aqueous layer was collected, and the PCI step repeated twice, followed by final chloroform/isoamyl alcohol treatment. DNA was precipitated with the addition of 1.0 mL ice-cold 99% (*v*/*v*) ethanol and 50 µL 3 M sodium acetate, followed by centrifugation (16,000× *g*, 5 min, 4 °C). The DNA pellet and washed with 70% (*v*/*v*) ethanol, air-dried, and resuspended with 50 µL 10 mM tris (pH 7.8) and stored at −20 °C until used. Host contamination was determined by amplification of partial 16S rDNA.

### 2.6. Sequencing of Phage Genome

Quality control, library preparation, and sequencing of extracted DNA from *Proteus*_virus_309 was performed by the Central Analytical Facility of Stellenbosch University. DNA quality was assessed for purity on the NanoDrop™ ND-1000 spectrophotometer (ThermoFisher Scientific), using a 1 x tris-EDTA buffer (ThermoFisher Scientific). The double stranded DNA (dsDNA) fraction was quantified on the Qubit 4.0 fluorometer (ThermoFisher Scientific) using the QubitTM 1× dsDNA HS assay kit according to the manufacturer’s protocol. MAN0017455 REVC.0 Library preparation was performed using the Ion Plus Fragment Library Kit (ThermoFisher Scientific) according to the manufacturer’s protocol, MAN0009847 revision K.0. Briefly, DNA was fragmented using the Covaris S2 focused ultrasonicator (Covaris, Inc.; Woburn, MA, USA) with a 10% duty cycle, 5% intensity, and 200 cycles/burst in two cycles; consisting of 60 s treatments each. Sheared DNA was purified using 1.8× volume Agencourt™ AMPure™ XP reagent (Beckman Coulter, Brea, CA, USA). The fragmented DNA was end-repaired at room temperature for 20 min and purified using 1.8× volume Agencourt™ AMPure™ XP reagent (Beckman Coulter). End-repaired DNA was blunt-end ligated to IonCode™ Barcode Adapters at 22 °C for 15 min and purified, using 1.4× volume Agencourt™ AMPure™ XP reagent (Beckman Coulter). The adapter-ligated libraries were amplified across nine polymerase chain reaction (PCR) cycles and assessed for fragment size distribution on the LabChip^®^ GX Touch 24 Nucleic Acid Analyzer, using the X-Mark DNA LabChip and HT DNA NGS 3K reagent kit (PerkinElmer, Waltham, MA, USA) according to the manufacturer’s protocol; CLS145098 Rev. F. The amplified libraries were combined in equimolar amounts and size-selected on the Pippen Prep (Sage Science, Beverly, MA, USA) using 2.0% dye-free gel cassettes with marker L, to retain DNA fragments of approximately 275 bp. The size selected library was purified, using 1.4× volume Agencourt™ AMPure™ XP reagent (Beckman Coulter). The combined library was quantified using the Ion Library TaqMan™ Quantitation kit (ThermoFisher Scientific) according to the protocol, MAN0015802, rev. C.0. Quantitative PCR was performed on the StepOnePlus™ Real-time PCR system (ThermoFisher Scientific). The combined library was diluted to a target concentration of 60 pM for template preparation using the Ion 540™ Chef kit (ThermoFisher Scientific). Twenty-five microliters of the diluted pooled library were loaded onto the Ion Chef™ (ThermoFisher Scientific) liquid handler for template preparation and enrichment using the Ion 540™ Chef reagent, solutions and supplies according to the protocol, MAN0010851 revision F.0. Enriched ion sphere particles were loaded onto an Ion 540™ chip (ThermoFisher Scientific). Massively parallel sequencing was performed on an Ion GeneStudio™ S5 Prime system using sequencing solutions and reagents (ThermoFisher Scientific) according to the manufacturer’s protocol, MAN0010851 revision F.0. Flow space calibration and BaseCaller analysis were performed using standard analysis parameters in the Torrent Suite version 5.16.0 software.

### 2.7. Genome Annotation and Analysis

The genome was assembled using CLC Genomics Workbench version 7.5.1 and default settings, except that both length and similarity fractions were adjusted to 0.9 (Qiagen, Hilden, Germany; RRID:SCR_011853). Annotation was performed with MultiPhate2 using protein sequences from the VOGDB (release 80), NCBI virus protein, PHANTOME and pVOGs databases accessed on 11 November 2021 [36]. Nucleotide-based intergenomic similarities between *Proteus*_virus_309 and related phages was calculated using VIRIDIC [37] and genome layout and synteny assessed using clinker [38]. Phylogenetic relationships to other phages were further investigated by determining protein cluster similarities between *Proteus*_virus_309 and all reported *Proteus* infecting viruses or those in the INSDC database that show significant nucleotide similarity using VirClust [39]. Bacphlip [40] was used to support the prediction of phage lifestyle (lytic or lysogenic).

### 2.8. Host Specificity Determination

Spot tests were performed on overlays seeded with three *P. mirabilis* isolates (B14, B15, B16), *P. mirabilis* ATCC 25933, and *P. vulgaris* B24. One hundred microliters of each overnight culture were inoculated into molten agar and poured on BHI agar (1.2%, *w*/*v*). Once plates solidified 10 µL of each high titer phage suspension (1 × 10^8^ PFU/mL, standardized) was spotted onto the overlay and incubated overnight at 37 °C, in triplicate. Lower concentrations of phage were used to determine plaque formation if zones of inhibition were observed.

### 2.9. Multiplicity of Infection (MOI) and One-Step Growth Curve Determination

Optimal MOI was determined using serial dilutions of *Proteus*_virus_309, inoculated with exponential phase *P. mirabilis* at different proportions of infection (1000, 100, 10, 1, 0.1, 0.01, 0,001). One hundred microliters of phage suspension, at various dilutions, and 100 µL of *P. mirabilis* were added to 5 mL of BHI (supplemented with 2 mM CaCl_2_) and incubated at 37 °C for 10 h. Cultures were centrifuged (8000× *g*, 10 min, 4 °C), the supernatant was harvested and filtered through nitrocellulose (0.2 µm) filters. Plaque forming units were determined by soft agar overlay (Figure S1).

A one-step growth curve was generated as described by Yin et al. [41] with modifications. A culture of *P. mirabilis* was grown up to approximately 1.0 × 10^8^ CFU/mL from which 1.0 mL was inoculated with 100 µL of *Proteus*_virus_309 at 1 × 10^7^ PFU/mL (MOI = 0.01). Incubation was for 5 min at 37 °C on an orbital shaker (100 rpm), cells were harvested (8000× *g*, 2 min), and the supernatant removed. Cells were resuspended in 1.0 mL BHI, transferred into 9.0 mL BHI, and incubated in a 37 °C water bath with constant agitation using a magnetic stirrer (100 rpm). Free phages were collected at 2.5 min intervals until 30 min (T_0_–T_30_) and at 5.0 min intervals for 50 min. Two hundred microliters were collected at each interval and 10 µL of chloroform was added, quickly vortexed, centrifuged (12,000× *g*, 1 min, 16 °C), and phage titers were determined. The assay was performed in triplicate, consecutively. Burst size was calculated as a ratio of free phage particles to the initial count of infected bacterial cells during the latency period.

### 2.10. Bacterial Lysis Assay and Phage Resistance

Bacterial lysis assay was determined as described by Yen et al. [42], but with some modifications. *Proteus*_virus_309 suspension (1 × 10^6^ PFU/mL) was added to a *P. mirabilis* (1 × 10^8^ CFU/mL) at an MOI of 0.01 and allowed to bind for 5 min. Bacterial cells were harvested (6000× *g*, 1 min, 16 °C), washed to remove free phage particles and resuspended in 1 mL of BHI. Infected bacteria were inoculated into 9.0 mL BHI and incubated at 37 °C on an orbital shaker (100 rpm). Viable bacteria were harvested every 15 min (T_0_–T_60_) for the first 60 min and every hour (T_60_–T_240_). Bacteria were serially diluted, plated onto BHI agar, and incubated at 37 °C for 16 h.

Phage resistant mutants were selected by exposing *P. mirabilis* B16 to *Proteus*_virus_309 (MOI = 0.01), incubated at 37 °C for 16 h. Selected mutants were streaked three times and DNA was extracted. PCR amplification of the of lipase gene (ORF 3, 633 bp) was used to screen for potential lysogeny with the following primers: Forward: 5′-GCAGTCGACGATAAAAAGTATGATACCCTCT-3′ and Reverse: 5′-TATGCGGCCGCCTATCTCTGTGTATCCTTG-3′ using 100 ng gDNA as template. An existing construct of pRSF_lipase was used as positive control (Figure S6).

### 2.11. Temperature and pH Stability

Temperature and pH stability tests were performed as described by Yang et al. [43]. Separate samples of 1.0 mL phage suspension (1 × 10^8^ PFU/mL) were incubated at 20, 37, 50, 60, 70, and 80 °C, respectively, for 1 h and PFU/mL determined. In a separate experiment, phage suspensions were inoculated into separate 5.0 mL BHI, pre-adjusted to pH 3–11, incubated for 3 h at 26 °C, and the PFU/mL determined. All experiments were performed in triplicate.

### 2.12. Statistical Analysis

The multiplicity of infection, one-step growth curves, bactericidal assay and stability assays were expressed as means and standard error of the means (SEM), and were determined using GraphPad Prism 5 (San Diego, CA, USA).

## 3. Results and Discussion

### 3.1. Isolation, Morphology and Host-Range Characterization

**Figure 1:**
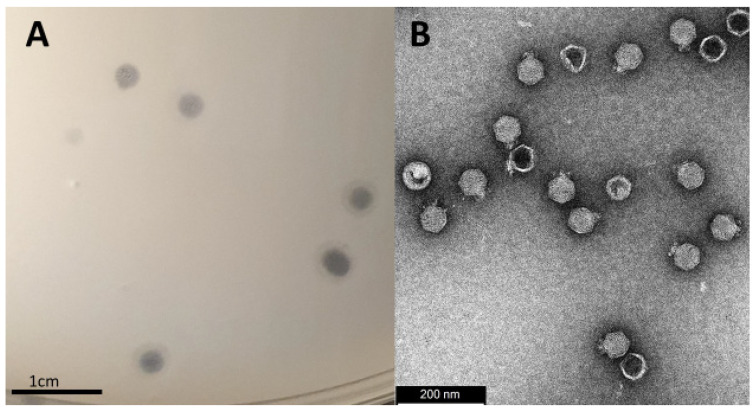
(**A**) Plaque morphology of *Proteus*_virus_309 showing a clear zone with a slight halo border. (**B**) Transmission electron micrograph of *Proteus*_virus_309 displaying icosahedral morphology with several damaged or empty viral particles represented by capsids with a darker center (less electron dense).

A clinical strain of *P. mirabilis* (B16) was used to isolate bacteriophages from a sewage sample. Evidence of host lysis was observed producing 4 mm clear plaques with a slight halo around the borders, as well as turbid plaques with no or very faint halos (Figure 1A). Transmission electron microscopy revealed that the phage has a short (18 nm) non-contractile tail and an icosahedral capsid of 67 nm in diameter (Figure 1B). Based on these results, *Proteus*_virus_309 belongs to the genus *Novosibovirus*. Other members of the genus have been identified in the United Kingdom and Russia [44,45]. Although host screening was not extensive, *Proteus*_virus_309 only infected the clinical isolate *P. mirabilis* B16 and showed no infectivity against the other *Proteus* species (Table S1). Three of the four phages closely related to *Proteus*_virus_309 (*Proteus* phages PM75, PM16 and RS8pmA) were also host-specific while phage RS1pmA infected two *P. mirabilis* strains. Phages PM85, PM93, and PM116 displayed a broader host range [44].

### 3.2. Physiological Characterization

**Figure 2:**
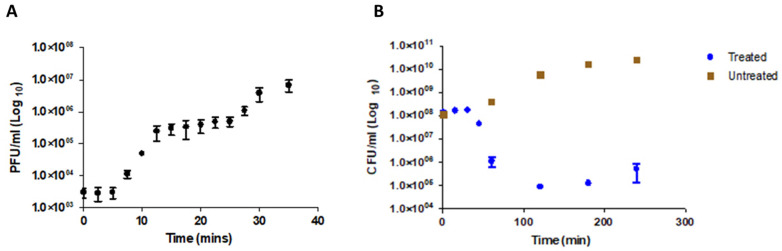
(**A**) One-step growth curve of *Proteus*_virus_309 and (**B**) lysis of *P. mirabilis* B16 culture following infection with phage 309 (MOI of 0.01). Lysis was followed over 4 h.

The one step growth curve (Figure 2A) revealed that *Proteus*_virus_309 has a short latency period of approximately 5 min and a rise period of 7.5 min. A burst was achieved after approximately 15 min, followed by a plateau of 15–20 min. This is rapid compared to lysis profiles of other *Novosibovirus* members, as listed in Table S2A,B, and reported in literature for *Proteus* phages [29,46,47]. Approximately 39 plaque forming units (PFUs) of *Proteus*_virus_309 were recorded per bacterial cell. This compared with the 32 PFUs per cell recorded for *Proteus* phage PM75 but is far less than the 100 PFUs per cell reported for *Proteus* phage PM16 [44]. *Proteus*_virus_309, at an MOI of 0.01, reduced the viability of a *P. mirabilis* B16 population by 99.9% (1.43 × 10^8^ log_10_ CFU/mL) within 120 min (Figure 2B). The short lytic cycle and overall bactericidal activity of bacteriophage 309 are promising attributes in the developing of bacteriophage therapy [48] or genetically engineered bacteriophages, as reported for *P. aeruginosa* phages [49]. Although *Proteus*_virus_309 is highly virulent, phage-resistant cells of *P. mirabilis* did develop after 16 h of incubation. This is a clear indication of a resistant subpopulation that became dominant once susceptible populations were predated by bacteriophage 309, a feature also noted for PM75 [44]. Plaque assays performed with phage-resistant isolates did not produce visible plaques, except one selected mutant C3, which produced a faint zone (Figure S5). Phase-variable expression of surface polysaccharides could be a likely mechanism of avoiding phage predation by generating a heterogenous population without the burden of mutations [50]. Phase variation has been observed in intestinal *Vibrio cholerae* whereby modulating the O1 antigen, important in host colonization, and avoiding predation by the O1 antigen-dependent lytic phage ICP1 [51]. These changes in bacterial fitness often have trade-offs, with such that avoiding phage predation can lead to less virulent variants becoming the dominant population. Although no similar relationships have been documented in *P. mirabilis*, phase-variable expression of fimbriae is common with many fimbrial phase variants being observed during colonization and biofilm development [52]. It is unknown if fimbriae and hemagglutinins produced by *P. mirabilis* are involved in phage infection and entry. Phage resistance can also be gained through other mechanisms such as spontaneous mutations, restriction modification systems, and CRISPR-Cas systems [53].

**Figure 3:**
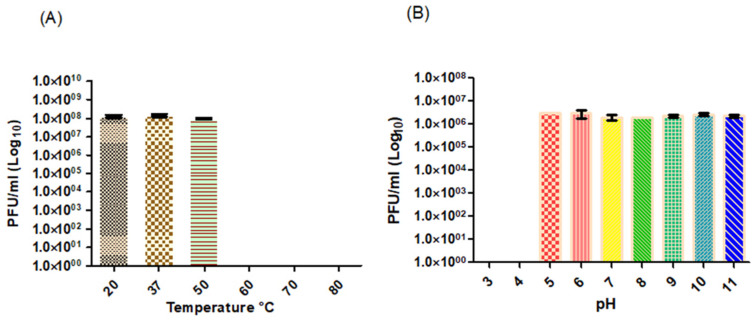
The effect of (**A**) temperature on phage stability, with no plaques detected >60 °C and (**B**) pH on phage stability, with no plaques detected at pH 3 and 4.

The thermal stability assay (Figure 3A) revealed that *Proteus*_virus_309 could not withstand temperatures above 50 °C. No significant differences (*p* < 0.05) were recorded in PFU/mL from 20 to 50 °C. Reduced titers of *Proteus* phages vB_PmiS-TH and vB_PmiS_PM-CJR were recorded after exposure to 70 °C [46,54], with complete inactivation at 80 °C. *Proteus*_virus_309 remained infective at pH values ranging from 5 to 11 (Figure 3B), with no significant difference observed across the range (*p* < 0.05). Complete inactivation was recorded at pH 4 and below (Figure 3B). *Proteus mirabilis* increases the pH of urine [5], which would favor phage stability.

### 3.3. Genome Characterization

**Figure 4:**
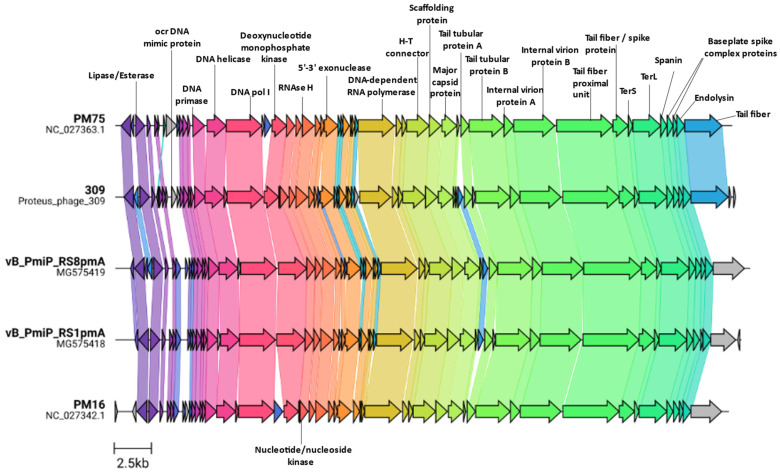
Comparison of *Proteus*_virus_309 genome with those of closely related viruses.

The genome of *Proteus*_virus_309 is 41740 bp, with a G + C content of 41.3%, thus slightly higher than the average G + C content of 39.0% recorded for *P. mirabilis* (Table S3). When compared against all *Proteus* infecting phages, *Proteus*_virus_309 is most related to vB_PmiP_RS8pmA (83.9%), vB_PmiM_RS1pmA (81.9%), *Proteus* virus PM16 (81.8%), and *Proteus* virus PM75 from the genus *Novosibovirus,* with 89.5% nucleotide similarity to the latter (Figure S2). Based on these results and the ICTV guidelines [55], *Proteus*_virus_309 is a member of the genus *Novosibovirus*. Comparison of all the *Novosibovirus* genomes described thus far shows that each represents a chimera of the others with several unique ORFs being present in some and absent in others (Figure 4). The lack of relation to *Proteus* phages other than those in the *Novosibovirus* genus is further supported by protein cluster analysis (Figure S2B). When comparing the top BLASTn hits of the *Proteus*_virus_309 against the Genbank database, two *Providencia* infecting phages (PSTRCR_114 and vB PstP PS3) showed highest similarity outside of *Novosibovirus* genomes. Protein cluster analysis further strengthened the relationship to these viruses through the identification of communal protein clusters resulting in the grouping of the *Providencia* phages in the same clade as those from *Novosibovirus* (Figure S3).

*Proteus*_virus_309 shares a unique open reading frame (ORF2) with *Proteus* phage vB_PmiP_RS8pmA, while more distantly related proteins are present in *Providencia* phage PSTRCR_114 and a range of *Krischvirus* species (RB49, JSE, Phi1 and ECD7). This ORF is divergently transcribed, and possibly co-regulated, from a putative lipase/esterase present on all *Novosibovirus* genomes. *Proteus*_virus_309 encodes a putative Ocr-like DNA mimic protein (ORF9), absent in all other classified *Novosibovirus*. The T7 Ocr protein protects viral DNA from digestion by host Type I restriction endonuclease by acting as a decoy [56] as well as interacting with the Bacteriophage exclusion (BREX) defense system [57]. The Ocr-like DNA mimic protein may have a similar role. The absence of this protein in other members of *Novosibovirus* suggests that those hosts do not target the phage DNA for degradation in the same way as for *Proteus*_virus_309 and its host *P. mirabilis* B16. The genomic region occupied by Ocr on the *Proteus*_virus_309 genome is occupied by a DNA adenine methylase (DAM) on *Proteus* phage PM75. These enzymes are multifunctional and could protect phage DNA from restriction endonucleases, play a role in promoter regulation or efficient release of progeny [58]. Modification of the genome at this position along with sharing a specific tail fiber protein with PM75 may partly explain the much larger burst size of PM16 compared with *Proteus*_virus_309 and PM75 [44]. This specific tail fiber protein (ORF57), shared only between PM75 and *Proteus*_virus_309 and no other known *Proteus* phages, may also explain the narrow spectrum host specificity of these two phages. In the other three *Novosibovirus* genomes, a conserved hypothetical protein is present at this location. For obligately lytic T-even phages, both the length of the latent period and the burst size increase if more phages adsorb to the cell when infected, termed lysis inhibition [59,60]. The latent period for PM16 was determined to be approximately 15 min, compared to more-or-less 20 min for PM75 [44]. Binding of phages before infection may be affected by tail fibers; however, at an MOI of 0.01 this mechanism may be ruled out. Additionally, PM16 lacks several small ORF’s between the exonuclease (ORF27) and the RNA polymerase (ORF35) present in *Proteus* virus 309 and PM75. Minor changes to the genome can substantially affect lysis timing and burst size, however, this does not account for changes in host physiology that assist, or are responsible for, larger burst sizes or increased latent periods.

### 3.4. Additional Features

The lipase/esterase mentioned here contains two known domains belonging to a family of SGNH_hydrolases and GDSL-like Lipase/Acylhydrolases of which the two families contain a diverse range of esterases and lipases, respectively. These hydrolases could play a role during infection through host adsorption or host lysis at the end of the lytic cycle [61,62]. Only some *P. mirabilis* strains acetylate their peptidoglycan and the degree of O-acetylation can differ markedly between strains [63,64,65]. Thus, if lipases and esterases are involved in host attachment, it may be strain-specific. Phages closely related to *Proteus*_virus_309 were all described as lytic viruses with no integrase and lysogens present [44,45,66]. Bioinformatic assessment of the *Proteus*_virus_309 genome using Bacphlip predicted that it is a lytic phage with a score of 0.99. Although phage resistant isolates were identified following infection (Figures S4 and S5), phages could not be detected, suggesting that these were not lysogens (Figure S6).

*Proteus*_virus_309 may not be a likely candidate for phage therapy due to its narrow host spectrum. Further research is required to determine if *Proteus*_virus_309 infection is limited to a specific host receptor or external host factors such a biofilm composition and capsule type [67]. Furthermore, whole genome sequencing of non-host genomes could elucidate internal host defenses, such as CRISPR-Cas systems, abortive infection, and restriction endonucleases, that are specific to 309-like phages and their prevalence across *Proteus* species preventing productive infection or re-infection. A recent insight into *P. mirabilis* revealed that Class I CRISPR-Cas systems belonging to type 1E were dominant across 109 *P. mirabilis* isolates [68].

## 4. Conclusions

*Proteus*_virus_309 is closely related to, but clearly distinguishable from, *Proteus* viruses PM75, vB_PmiP_RS8pmA, vB_PmiM_RS1pmA and *Proteus* virus PM16 and, therefore, belongs to the *Novosibovirus* genus. Similar, to other *Novosibovirus* members, *Proteus*_virus_309 has a narrow spectrum of activity, infecting only *P. mirabilis* B16 isolated from a urinary tract infection. Although 309 is highly strain-specific leading to it likely to be ignored as a candidate for phage therapy on its own, it may be combined with other *Novosibovirus* and more broadly infecting lytic *Proteus* phages into a *Proteus*-specific phage cocktail. Such a cocktail could be effective in treating CAUTI’s, wound infections if topically applied, used as disinfectant, or as a prophylactic coating of urine catheters. The ability of *Proteus*_virus_309 to destruct biofilms formed by *Proteus* is currently being investigated.

## Data Availability

The genome of *Proteus*_virus_309 is available of the GenBank database under accession number: OL416096.

## Supplementary Materials

The following supporting information is available online at https://www.mdpi.com/article/10.3390/v14061309/s1, Table S1: *Proteus*_virus_309 host specificity and identification based on 16s rDNA sequences. Table S2A: Comparison of physiological characteristics of *Novosibovirus* members. Table S2B: Comparison of the lysis profiles of *Proteus* infecting phages. Table S3: Sequencing analysis report for the assembly of phage *Proteus*_virus_309. Figure S1: The effects of different MOIs on the production of phage production. A one-way ANOVA showed significant difference between an MOI of 0.01 and MOI’s ≥0.1 with no significant difference between 0.01 and 0.001 with higher titer lysates produced from MOI = 0.01. Figure S2: (A) Intergenomic similarity between all *Proteus* infecting phages determined using VIRIDIC. *Proteus*_virus_309 is highlighted with a red border. Continued: (B) Protein cluster clustering between all *Proteus* infecting phages determined using VirClust. Figure S3: Protein cluster clustering (a) between all phage that show significant nucleotide similarity to *Proteus*_virus_309 determined by VirClust. Figure S4: (A) Dienes phenomenon observed across different strains of *Proteus mirabilis* unrecognizable to self, as *P. mirabilis* B16 does form a dienes line between the *P. mirabilis* ATTC 25933. The phage resistant mutants (C1–C4) that arose after the bactericidal assay are still recognized by the mother strain (phage-unexposed). Figure S5: (A) is a positive spot test of colony 3. (B) is the spot assay of the *P. mirabilis* B16 (used to generate the mutants). (C) is Colony 4, a phage resistant mutant. Notable that colonies C1, C2, and C4 are all resistant to the phage whilst a cloudy zone appears for C3. Figure S6: PCR assay for the presence of lipase gene (ORF 3, 633 bp) in host DNA to determine lysogeny. From left to right: Lambda PstI ladder; Lane 1 negative control; Lane 2–5 PCR amplicons from phage resistant mutants (C1–C4); Lane 6 pRSF_lipase construct positive control; Lane 7–10 One microliter of gDNA of each of the mutants; Lane 11 contains 1 µL of the pRSF_lipase construct.
