# Supplementary material for: Isolation and Characterization of Lytic Proteus Virus 309
Source: Viruses. 2022 Jun 15;14(6):1309. doi: 10.3390/v14061309 (PMC9229222; doi:10.3390/v14061309)
Supplement: Supplementary file 1 [file viruses-14-01309-s001.zip › viruses-1744158-supplementary.pdf]

Table S1: *Proteus*_virus_309 host specificity and identification based on 16s rDNA sequences

Isolate	Top two BLASTn hits	Coverage%	Identity%	Accession	Source	Infectivity
P. mirabilis B16	*Proteus mirabilis* strain MPE4069 chromosome, complete genome	100	100	CP053718.1	Human urine isolate (Tygerberg Hospital)	+
	Proteus mirabilis strain P8 16S ribosomal RNA gene, partial sequence	100	100	MT276305.1		
P. mirabilis B15	*Proteus mirabilis* strain MPE5203 chromosome, complete genome	99	100	CP053685.1	Human urine isolate (Tygerberg Hospital)	-
	Proteus mirabilis strain AGMDRUC5 16S ribosomal RNA gene, partial sequence	99	100	MN795608.1		
P. mirabilis B14	*Proteus mirabilis* strain 1701092 chromosome, complete genome	100	100	CP042857.1	Human urine isolate (Tygerberg Hospital)	-
	Proteus mirabilis strain VS-26 16S ribosomal RNA gene, partial sequence	100	100	MK785117.1		
P. vulgaris B24	*Proteus vulgaris* strain EX14 16S ribosomal RNA gene, partial sequence	100	99,85	MT066063.1	Human urine isolate (Tygerberg Hospital)	-
	Proteus vulgaris strain FDAARGOS_556 chromosome, complete genome	100	99,78	CP033736.1		
P. mirabilis ATCC 25933					American Type Culture Collection	-
E. coli B8	*Escherichia coli* strain EC 16S ribosomal RNA gene, partial sequence	100	99,78	MN083306.1	Human urine isolate (Hospital)	-

	Escherichia coli strain LWY24 chromosome, complete genome	100	99,71	CP054556.1

Table S2A: Comparison of physiological characteristics of *Novosibovirus* members

Phage	Susceptible Hosts	Latent period (min)	Plaque forming units per cell	Source	Reference
309	*P. mirabilis* strain B16	5	39	Cape Town, South Africa	Current study
PM16	*P. mirabilis* strain CEMTC 73	15	~100	Novosibirsk, Russia	[4]
PM75	*P. mirabilis* strain CEMTC 821	20	32	Novosibirsk, Russia	[4]
vB_PmiP_RS1pmA	*P. mirabilis* strain RS1 & RS3	unavailable	unavailable	United Kingdom	[4]
vB_PmiP_RS8pmA	*P. mirabilis* strain RS8	unavailable	unavailable	United Kingdom	[4]

Table S2B: Comparison of the lysis profiles of *Proteus* infecting phages

Phage	Burst (min)	Plaque forming units per cell	Reference
309	15	39	Current study
vB_PmiP_5460	10-15	46	[29]
vB_PmiM_5461	25	11	[29]
vB_PmiS-TH	20	260	[46]
Privateer	40	140	[47]
PM85	7	18	[44]
PM93	7	75	[44]
PM116	7	70	[44]

Table S3: Sequencing analysis report for the assembly of phage *Proteus*_virus_309

Feature	Value
Total number of reads	9909485
Average Read length (bp)	194.49
Genome size (bp)	41740
DNA G+C (%)	41.3
Average Coverage	45595x
Number of ORFs	59
Accession number	OL416096
Bacphlip virulence score	0.99
Highest percent nucleotide similarity with a species in the same genus	89.5
Lineage	*Duplodnaviria, Heunggongvirae, Uroviricota, Caudoviricetes, Caudovirales, Autographiviridae, Slopekvirinae, Novosibovirus*

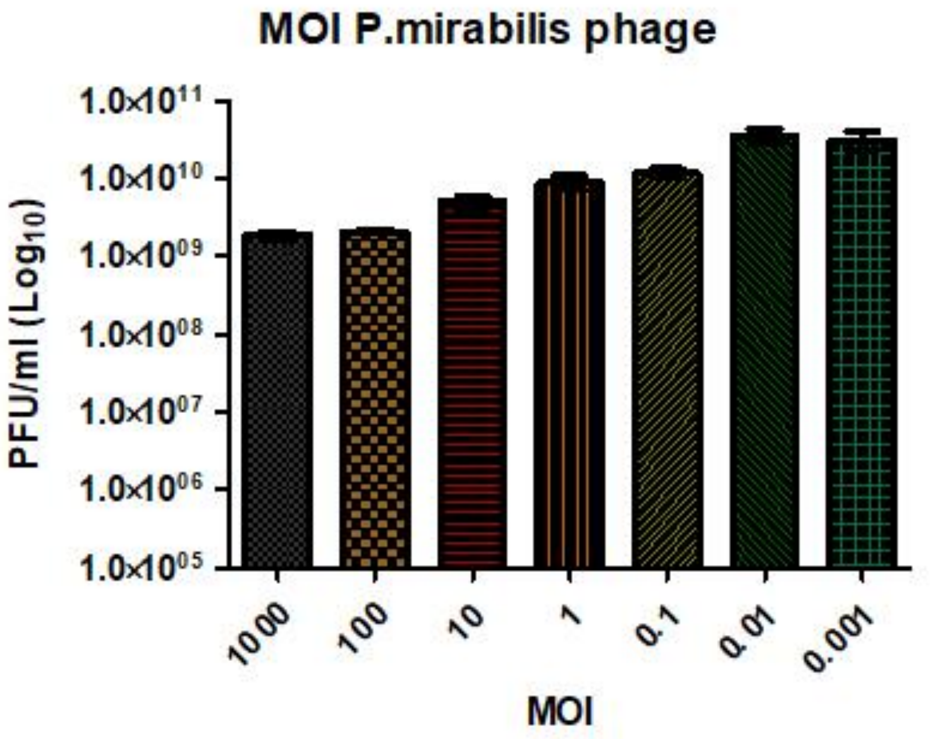

Figure S1: The effects of different MOIs on the production of phage production. A one-way ANOVA showed significant difference between an MOI of 0.01 and MOI's ≥0.1 with no significant difference between 0.01 and 0.001 with higher titre lysates produced from MOI= 0.01

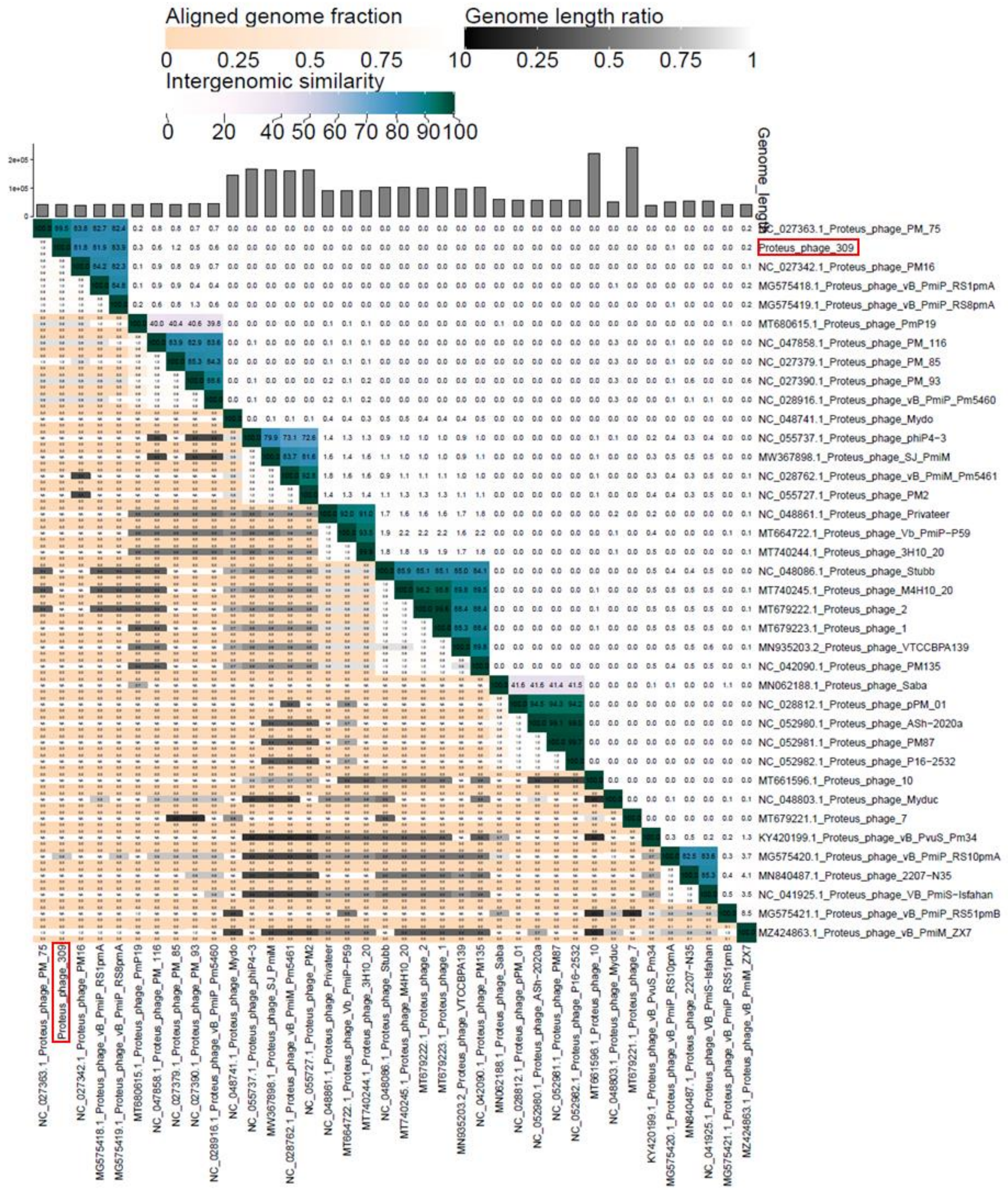

Figure S2: A) Intergenomic similarity between all *Proteus* infecting phages determined using VIRIDIC. *Proteus*_virus_309 is highlighted with a red border.

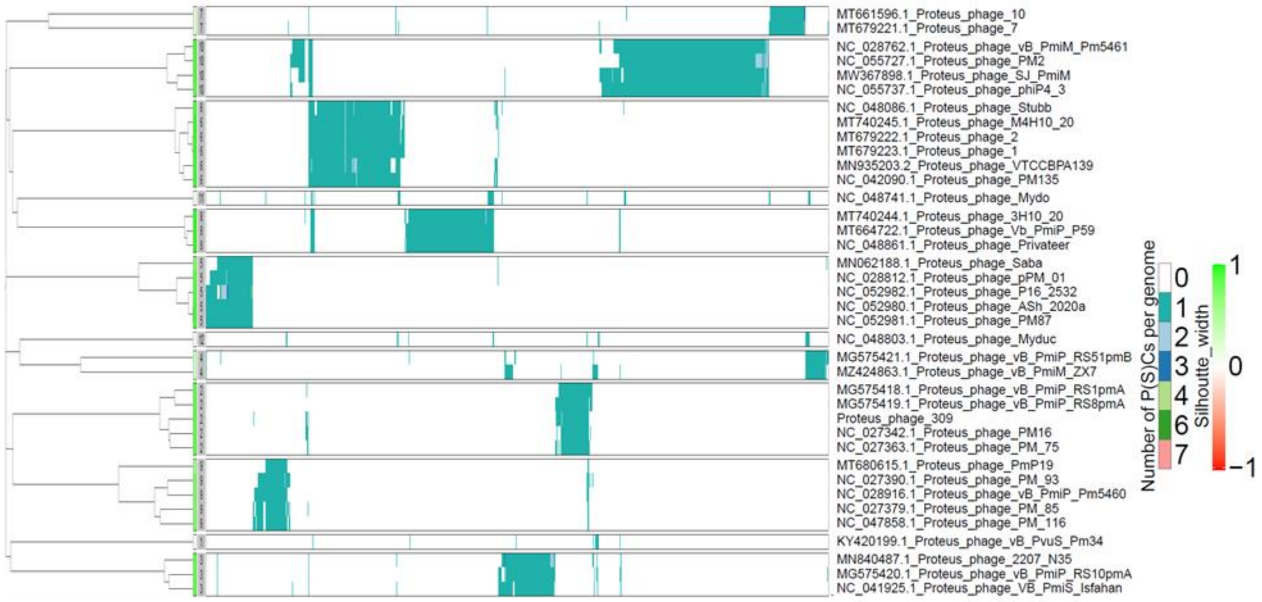

Figure S2 continued: B) Protein cluster clustering between all *Proteus* infecting phages determined using VirClust

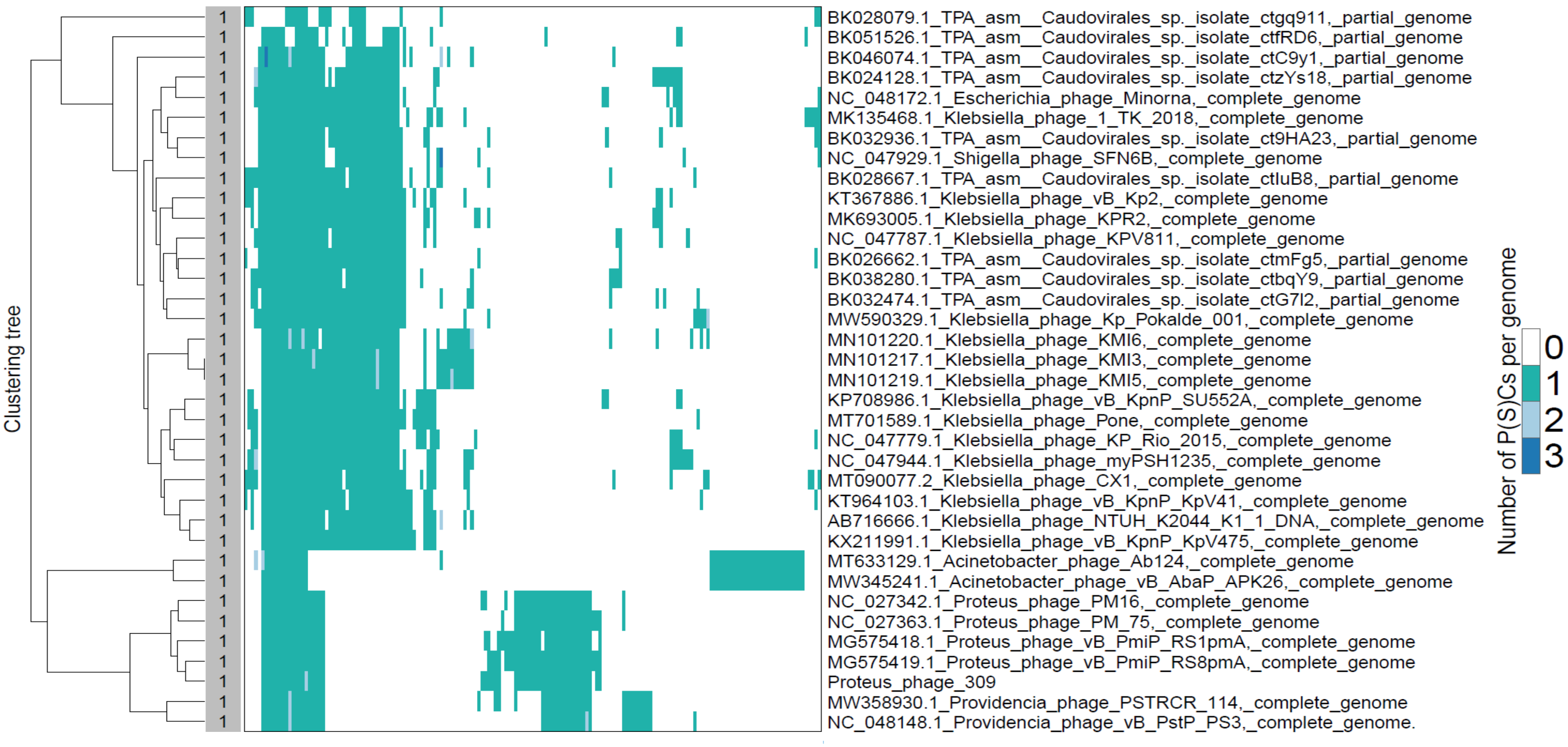

Figure S3: Protein cluster clustering (a) between all phage that show significant nucleotide similarity to *Proteus*virus309 determined by VirClust

.

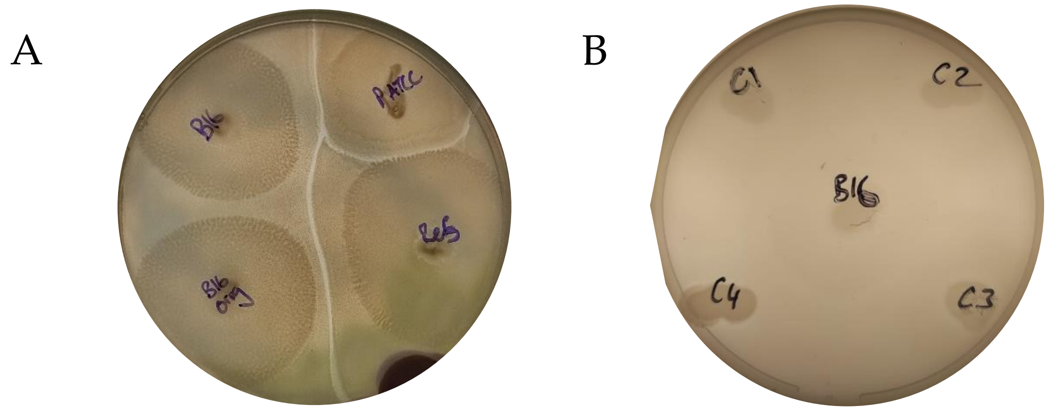

Figure S4: A) Dienes phenomenon observed across different strains of *Proteus mirabilis* unrecognisable to self, as *P. mirabilis* B16 does form a dienes line between the *P. mirabilis* ATTC 25933. The phage resistant mutants (C1-C4) that arose after the bactericidal assay are still recognized by the mother strain (phage-unexposed).

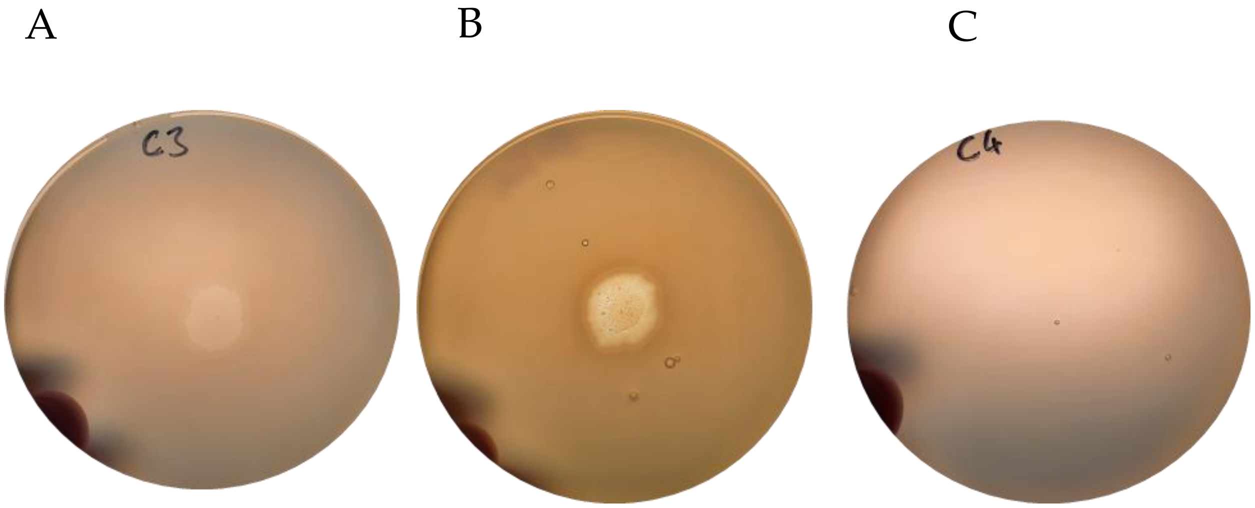

Figure S5: A) is a positive spot test of colony 3. B) is the spot assay of the *P. mirabilis* B16 (used to generate the mutants). C) is Colony 4 a phage resistant mutant. Notable that colonies C1, C2 and C4 are all resistant to the phage whilst a cloudy zone appears for C3.

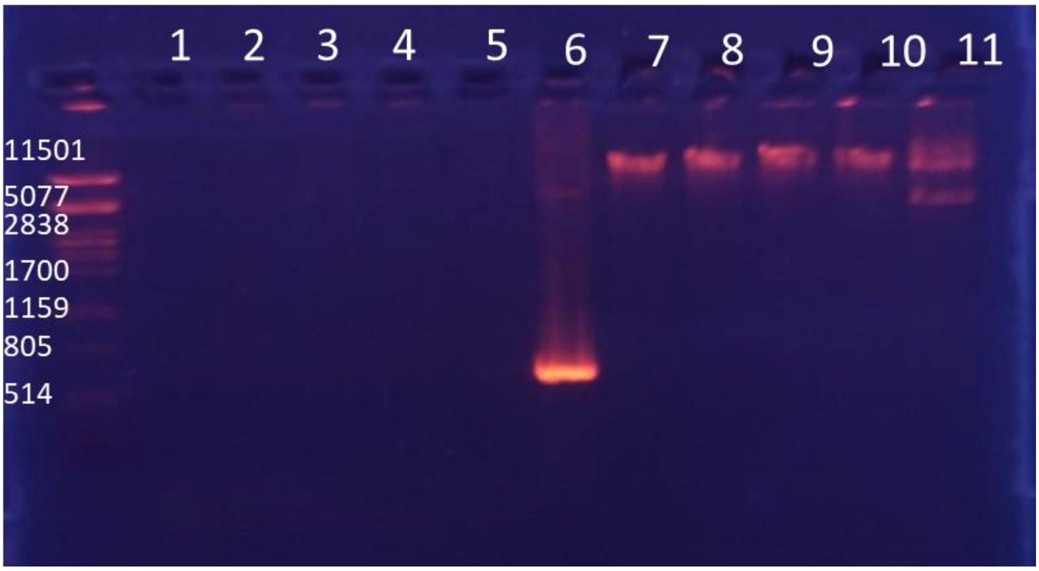

Figure S6: Figure S6: PCR assay for the presence of lipase gene (ORF 3, 633bp) in host DNA to determine lysogeny. From left to right: Lambda PstI ladder; Lane 1 negative control; Lane 2-5 PCR amplicons from phage resistant mutants (C1 - C4); Lane 6 pRSF_lipase construct positive control; Lane 7-10 One microliter of gDNA of each of the mutants; Lane 11 contains 1μl of the pRSF_lipase construct
